# Evidence-Based Policy on Deworming

**DOI:** 10.1371/journal.pntd.0000359

**Published:** 2009-01-27

**Authors:** Dirk Engels, Lorenzo Savioli

**Affiliations:** Department of Neglected Tropical Diseases, World Health Organization, Geneva; *PLoS Neglected Tropical Diseases*, United States of America

We read with interest “Does Deworming Improve Growth and School Performance in Children?” (published in this issue [1]), a summary of the 2007 Cochrane systematic review by David Taylor-Robinson and colleagues. Their previous systematic review [2], published in the *BMJ*, has been subject to substantial criticism by various authors and institutions [3]–[7]. Taylor-Robinson and colleagues have now responded to these criticisms by updating the original Cochrane review to include a number of recent trials and by giving more attention to two of the previous criticisms, i.e., outcome after longer follow-up and additional analysis taking worm intensity and prevalence into account.

We believe, however, that the current updated review remains limited in scope and does not bring substantially more value to their first systematic review, published in 2000 [1]. Our uneasiness with the updated review continues to reside in the use of a clinical epidemiological approach applied to public health and policymaking, and the fallacies that such an approach is likely to bring about.

It is worth noticing in this respect that another extensive review and meta-analysis—including more studies, of which most are overlapping with those included in the Cochrane review—was published earlier this year [8]. This review presents much more conclusive evidence in favour of systematic deworming, and also dedicates a section to a comparison with the Cochrane review conclusions. We believe that the more firm conclusions by Hall et al. are essentially due to (a) a more cautious consideration of the particular aspects of the transmission dynamics of intestinal helminths; (b) the multi-factorial origin of improvements to which deworming contributes; and (c) the cost–benefit and public health policy rationale on which the WHO recommendation in favour of systematic deworming is based. Public health policy setting indeed has to go beyond the issue of whether a sacrosanct level of statistical significance is reached on a limited number of (multi-factorial) outcomes in a series of carefully selected studies.

The World Health Organization (WHO) is currently promoting the large-scale implementation of “preventive chemotherapy”—i.e., the use of anthelminthic drugs—at various intervals, either alone or in combination depending on disease endemicity—as a public health tool for preventing morbidity due to infection with more than one helminth at a time [9]. Polyparasitism is indeed the rule more than an exception in poor settings. The preventive chemotherapy approach should logically have a very much enhanced impact at a minimal increase in cost and should ease the concerns of the Cochrane Collaboration as to whether large-scale deworming is worthwhile or not. We therefore look forward to a first Cochrane systematic review evaluating the comprehensive impact of deworming in such a multi-disease perspective.
